# Lacrimal gland abscess presenting with preseptal cellulitis depicted on CT

**DOI:** 10.1186/s12348-015-0068-6

**Published:** 2016-01-12

**Authors:** Daniel Thomas Ginat, Lora Rabin Dagi Glass, Fatoumata Yanoga, Nahyoung Grace Lee, Suzanne K. Freitag

**Affiliations:** Department of Radiology, University of Chicago, Pritzker School of Medicine, 5841 S Maryland Avenue, Chicago, IL 60637 USA; Department of Ophthalmology, Massachusetts Eye and Ear Infirmary, Harvard Medical School, Boston, MA USA; Department of Ophthalmology, University of Chicago, Pritzker School of Medicine, Chicago, USA

**Keywords:** Lacrimal gland abscess, Bacterial, Cellulitis, CT

## Abstract

**Background:**

Pyogenic lacrimal gland abscesses are uncommon and thus may not be immediately clinically recognized without a high index of suspicion.

**Findings:**

We present two patients with preseptal cellulitis and characteristic low-attenuation fluid collections in the lacrimal glands demonstrated on computed tomography (CT).

**Conclusions:**

Lacrimal gland abscesses should be considered when dacryoadenitis is refractory to medical treatment. Indeed, these cases highlight the value of prompt recognition of lacrimal abscess through ophthalmologic referral and the use of diagnostic imaging. Both patients were successfully treated via incision and drainage.

## Findings

### Introduction

Lacrimal gland bacterial abscesses are uncommon and may arise in the setting of acute dacryoadenitis, which may in turn develop secondary to an adjacent infection, such as rhinosinusitis, from hematogenous spread of bacteremia or after trauma [1, 2]. We present two patients with lacrimal gland abscess presenting with preseptal cellulitis depicted on computed tomography (CT).

### Case reports

**Fig. 1:**
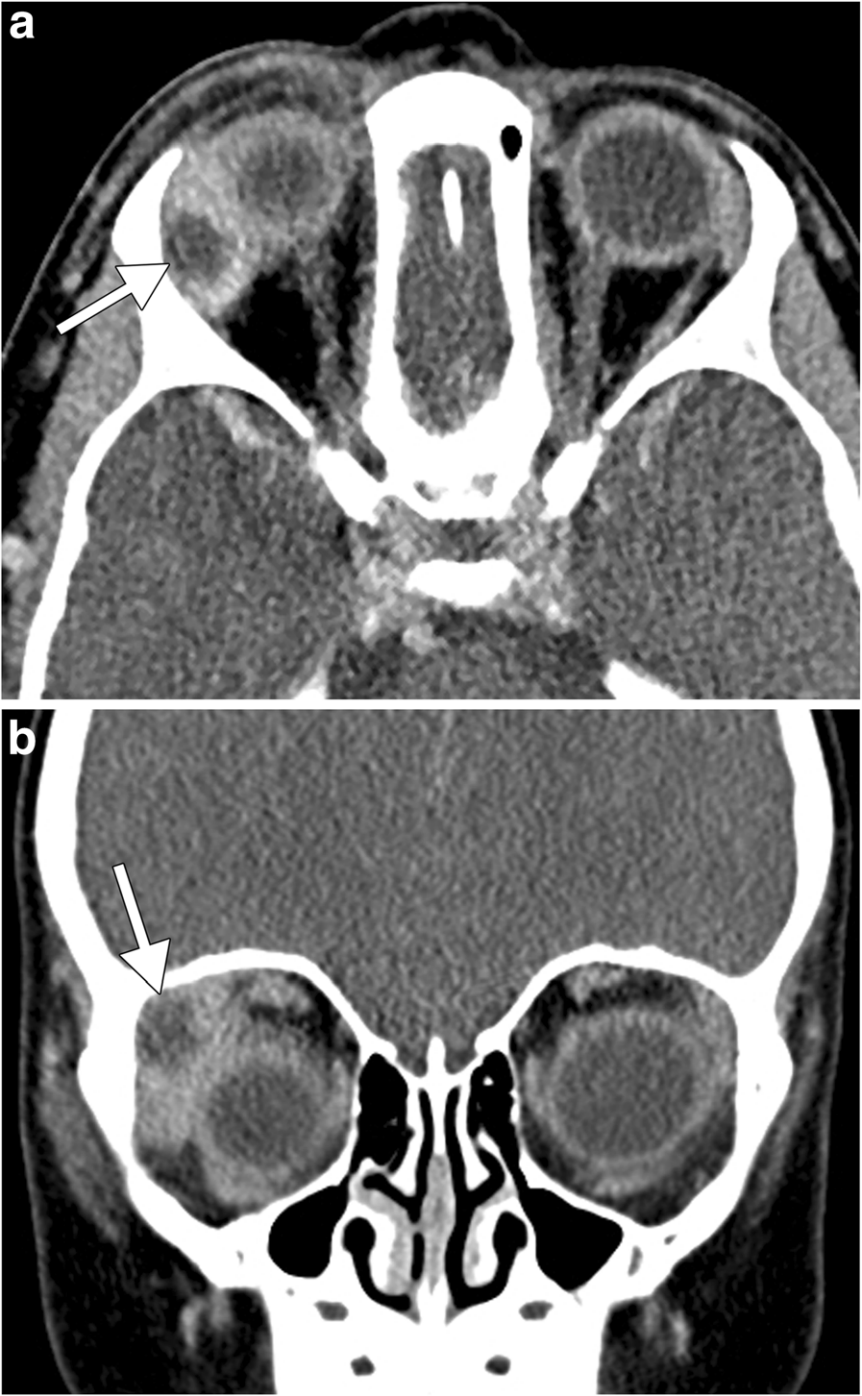
Axial (**a**) and coronal (**b**) contrast-enhanced CT images demonstrate right preseptal swelling and fat stranding as well as swelling and hyperenhancement of the right lacrimal gland, which contains a fluid collection (*arrow*)

Patient 1. The patient is a 2-year-old male who presented with 8 days of right eyelid swelling. Initially, the patient’s primary care physician diagnosed preseptal cellulitis and gave a 5-day course of oral clindamycin 15 g/ml BID. The eyelid swelling initially decreased after initiation of the antibiotics, but after 3 days, the right eyelid swelling increased to the point that the patient had nearly complete ptosis. The patient was then referred to ophthalmology whereby an examination revealed right preseptal erythema and swelling, conjunctival injection and chemosis, and decreased abduction. There was no afferent pupillary defect in the affected eye. A contrast-enhanced CT of the orbits was obtained, which showed right preseptal swelling, as well as marked enlargement of the right lacrimal gland with an area of central low attenuation, and swelling of the adjacent extra-ocular muscles (Fig. 1). An orbitotomy with drainage was performed with culture of purulent abscess contents. The cultures grew methicillin-resistant *Staphylococcus aureus* susceptible to sulfamethoxazole/trimethoprim. After completing a 3-week course of sulfamethoxazole (40 mg)/trimethoprim (8 mg) BID PO and tobramycin ointment, the patient’s symptoms had resolved.

**Fig. 2:**
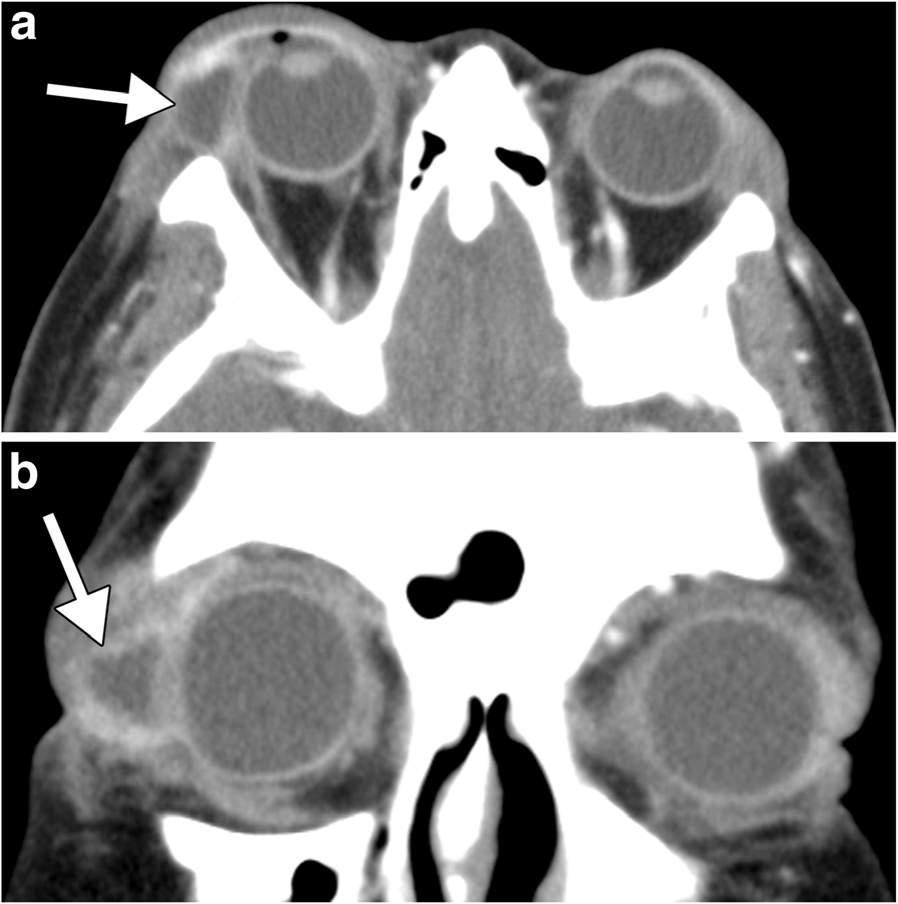
Axial (**a**) and coronal (**b**) contrast-enhanced CT images demonstrate right preseptal swelling and fat stranding and a fluid collection within the lacrimal gland (*arrow*)

Patient 2. The patient is a 60-year-old female with a past medical history of asthma and hypertension who presented with right upper lid swelling and pain for 4 days, purulent discharge, and limited supraduction and abduction of the right eye. A contrast-enhanced CT demonstrated right preseptal cellulitis, lacrimal gland swelling, and fluid collection (Fig. 2). An initial eye swab culture yielded abundant diphtheroids and *S. aureus* susceptible to sulfamethoxazole/trimethoprim. Incision and drainage of the abscess was subsequently performed, and the patient was discharged on polymyxin B sulfate and trimethoprim ophthalmic solution and PO sulfamethoxazole (800 mg)/trimethoprim (160 mg) BID for 3 weeks, which led to resolution of the infection.

### Discussion

Although pyogenic lacrimal gland abscesses are rare, these lesions are of clinical significance since, as in this case, the abscess may not improve with medical therapy alone and may require surgical drainage. Furthermore, if inadequately treated, patients may progress to more widespread and sometimes life-threatening involvement including intracranial abscesses, meningitis, and cavernous sinus thrombosis [3].

Radiological imaging plays an important role in the evaluation of complicated orbital infections [3, 4]. Both CT and magnetic resonance imaging (MRI) of the orbits and brain with contrast are valid imaging modalities for the evaluation of intraorbital abscesses and associated intracranial abnormalities. Technique with low ionizing radiation dose can be implemented, particularly for pediatric patients, without significantly compromising diagnostic performance, as in case of patient 1. Imaging may also be useful for detecting potential predisposing factors, such as rhinosinusitis and lacrimal gland ductal cyst [1, 2].

Lacrimal gland abscesses appear as characteristic low-attenuation areas within an enlarged lacrimal gland on CT [5]. There can also be diffuse enlargement and hyperenhancement of the affected lacrimal gland parenchyma surrounding the abscess. There is frequently associated orbital cellulitis. MRI with diffusion-weighted imaging can demonstrate restricted diffusion within the abscess. Nevertheless, the differential diagnosis for lacrimal abscess on imaging may include lacrimal gland tumors, lymphoproliferative disorders, foreign body granulomas, and sarcoidosis.

In conclusion, it is essential to recognize the presence of lacrimal gland abscesses, since these require incision and drainage. Therefore, diagnostic imaging of the orbits should be performed to evaluate for an underlying abscess in cases of medically refractory or atypical dacryoadenitis.

## Abbreviations

CT: computed tomography
MRI: magnetic resonance imaging
